# *Macleaya cordata* Extract Decreased Diarrhea Score and Enhanced Intestinal Barrier Function in Growing Piglets

**DOI:** 10.1155/2016/1069585

**Published:** 2016-07-25

**Authors:** Gang Liu, Guiping Guan, Jun Fang, Yordan Martínez, Shuai Chen, Peng Bin, Veeramuthu Duraipandiyan, Ting Gong, Myrlene Carine B. Tossou, Naif Abdullah Al-Dhabi, Yulong Yin

**Affiliations:** ^1^Key Laboratory of Agro-Ecological Processes in Subtropical Region, Institute of Subtropical Agriculture, Chinese Academy of Sciences, Hunan Provincial Engineering Research Center of Healthy Livestock, Scientific Observing and Experimental Station of Animal Nutrition and Feed Science in South-Central China, Ministry of Agriculture, Hunan Co-Innovation Center of Animal Production Safety, Hunan 410125, China; ^2^College of Bioscience and Biotechnology, Hunan Agricultural University, Changsha, Hunan 410128, China; ^3^Study Center of Animal Production, Faculty of Veterinary Medicine, University of Granma, Bayamo, Carretera Manzanillo 17 km, P.O. Box 21, 85100 Granma, Cuba; ^4^Department of Botany and Microbiology, Addiriyah Chair for Environmental Studies, College of Science, King Saud University, P.O. Box 2455, Riyadh 11451, Saudi Arabia; ^5^Chongqing Medical and Pharmaceutical College, Shapingba, Chongqing 401331, China

## Abstract

*Macleaya cordata* extract is of great scientific and practical interest to researchers, due to its antimicrobial and anti-inflammatory responses within experimental animals. This study was designed to determine the diarrhea score and innate immunity of growing piglets after they had received* Macleaya cordata* extract supplements. A total of 240 growing pigs were randomly assigned to one of three dietary treatments, with 8 replicates per treatment and 10 piglets per replicate. All pigs received a basal diet containing similar amounts of nutrients. The three treatments were a control (no additive), an antibiotic (200 mg/kg colistin), and the* Macleaya cordata* extract supplement group (40 mg/kg* Macleaya cordata* extract). The diarrhea score was calculated after D 28. The jejunal samples were obtained from five piglets selected randomly from each treatment on D 28. In comparison with the control group, the dietary* Macleaya cordata* extract and colistin group demonstrated a substantially decreased diarrhea score. The introduction of* Macleaya cordata* extract supplements to the diet significantly increased volumes of ZO-1 and claudin-1, particularly in comparison with the pigs in the control group (*P* < 0.05). The findings indicate that* Macleaya cordata* extract does enhance intestinal barrier function in growing piglets and that it could be used as a viable substitute for antibiotics.

## 1. Introduction

In recent years, there has been an increased focus on reducing or eliminating the use subtherapeutic antibiotics within livestock diets. For this reason, there has been a concerted effort to identify alternative effective therapies that could serve as a substitute for antibiotics in pig diets. The aim is to maintain acceptable performance levels and ensure food safety for consumers [1]. The medicinal plant extracts used as an alternative for antibiotics in swine diets are effective when it comes to increasing BW, intestinal health, nutrient digestibility, and antioxidative potential, and immunity. They also decrease the incidence and vulnerability to diarrhea [1, 2].

*Macleaya cordata* extract is believed to be an effective natural appetiser in swine, bovine, poultry, and even fish nutrition [3].* Macleaya cordata* extract is blended from intact aerial parts and a fraction of quaternary benzo[c]phenanthridine alkaloids (QBAs), primarily sanguinarine (SG) and chelerythrine (CH), and standardised to 1.5% w/w SG [4].

Sanguinarine (SA), a quaternary benzo[c]phenanthridine alkaloid, is synthesised from dihydrosanguinarine, via the activities of dihydrobenzophenanthridine oxidase [5]. It has been shown to have beneficial effects. It is antimicrobial [6, 7], antifungal, adrenolytic, sympatholytic, immunomodulatory [5], and anti-inflammatory [8, 9].

Chelerythrine can be found within the greater celandine plant and a number of additional poppy* Fumaria* varieties. It is a quaternary benzo[c]phenanthridine alkaloid. According to studies, it mostly exhibits tumour resistant, microbe resistant, and inflammation resistant qualities. Plus, the substance is a powerful disruptor when it comes to PKC (or protein kinase C). As such, the prospective utilisation of CHE, as a form of inflammation resistance, has been the topic of much debate. Its qualities are linked to its capacity to engage with DNA and proteins [10]. This is an enzyme that plays an important part in the control of signal transduction, cell propagation, and cell variation [11].

For this study, we hypothesised that dietary supplementation with* Macleaya cordata* extracts might stimulate the immune system and, as a consequence, decrease the incidence of diarrhea in growing piglets. Thus, the objective of the study was to evaluate the impact of* Macleaya cordata* extract, as a dietary supplement, on diarrhea scores and the expression of tight junction proteins.

## 2. Materials and Methods

### 2.1. Experimental Design, Animals, and Housing

All of the procedures used in this study have been approved by the Institute of Subtropical Agriculture, the Chinese Academy of Sciences Animal Care and Use Committees. A total of 240 (Yorkshire × Landrace) × Duroc (56 d old; BW 15.82 ± 1.13 kg) pigs were obtained from a commercial swine herd in Guangdong, China. 240 piglets were assigned, in completely random design (but based on their BW), to 24 pens. The pens each contained a total of ten piglets. The process was carried out according to the experimental animal allotment program created by Kim et al. [12]. All pens were equipped with a feeder, a nipple-type drinker (with modification; more details later), and plastic-covered expanding metal floors. The room temperature was set at 28.0°C and slowly increased by 0.5°C/wk.

All piglets were fed the same basal diet, with a similar amount of nutrients (Table 1). This diet was formulated to meet or exceed the NRC (2012) nutrient specifications for pigs weighing 10 to 30 kg. In total, there were 3 experimental treatments and study groups: the negative control (NC; no in-feed or in-water additive), the antibiotic group (AB; in feed, 0.2 g/kg colistin), and the* Macleaya cordata* extract group (MC; in-feed, 40 mg/kg* Macleaya cordata* extract). The* Macleaya cordata* extract was kindly provided by Phytobiotics Futterzusatzstoffe GmbH, Eltville, in Germany and Micolta Bioresource Inc., in Changsha, China. The pigs had unlimited access to feed and water throughout the entirety of the study.

### 2.2. Sample Collection and Analytical Procedure

On days 7, 21, and 28 of treatment, 10 mL of blood was collected in plastic uncoated tubes between 8:00 and 10:00 a.m. After collection, these blood samples were centrifuged at 8000 ×g for 10 min at 4°C, with sera collected and frozen at −20°C until further analyses.

### 2.3. Faecal Score and Sampling

To evaluate diarrhea incidence, faecal consistency scores (0, normal; 1, soft faeces; 2, mild diarrhea; and 3, severe diarrhea) were determined for each pen. These scores were collected by a trained individual, but who had no prior knowledge of the dietary treatments provided. The incidence of diarrhea was calculated by dividing the total number of pigs with diarrhea by the total number of all experimental pigs. The rate ratios were then calculated. On day 28 of the experiment, a number of the pigs (1 pig/pen) were killed. 1 sample of jejunal mucosa was collected in a sterile sample bag and stored at −20°C until analysis.

### 2.4. Concentration of IgG, IgM, D-Lactate, DAO, and TNF-*α*

The serum volumes of IgM and IgG were calculated according to readings from the 1251 Radio Immunoassay Analysing tool (Beijing North Institute of Biological Technology, Beijing, China). A *γ*-calculating instrument GC-300 (Zhongjia Co., Ltd., Beijing) was also used as part of this equipment. Recommendations from the maker were followed at all times.

The volume of serum D-lactate and DAO were calculated using Beckman Cx4 Chemistry Analyser (Beckman Coulter, Brea, CA) with a kit (Nanjing Jiancheng Bioengineering Institute, Nanjing, Jiangsu, China). Recommendations from the maker were followed at all times. All changes to or within the serum diamine oxidase were calculated with assay equipment.

The proportion of tumour necrosis factor-*α* (TNF-*α*) within serum was calculated via the use of a Porcine TNF-*α* Colorimetric ELISA tool (Nanjing Jiancheng Bioengineering Institute, Nanjing, Jiangsu, China). 50 *μ*L of regular plus dilute solution (or 100 *μ*L of the sample) was introduced to microplate wells. The wells had already been pretreated with capture antibody, as well as biotinylated antibody reagent. Identification relied on the utilisation of horseradish peroxidase, a stop solution of 0.18 N H_2_SO_4_, and TMB substrate. The identification parameter for TNF-*α* was 5 pg/mL. Merging was detected at 450 and 540 nm, with the use of the KC4 data evaluation programme and an ELISA plate tool.

### 2.5. Tight Junction Proteins Expression

The tight junction protein displays of occludin, claudin-1, and zonula occludens-1 (ZO-1) were calculated with the use of western blotting. Western blot testing was carried out via the use of a sophisticated chemiluminescence identification tool (Amersham, Illinois). The results were captured with the ChemiScope 3400 device (Clinx Science Instruments, Shanghai, China). They were evaluated with the use of Quantity One equipment (Bio-Rad, California, USA). The *β*-actin functioned as an interior control because it expressed no dissimilarities across the populations. The significance of the protein display was represented by the ratio of the densitometry units of *β*-actin and tight junction protein. For a short time, all protein was removed, according to the advice provided by a protein removal tool (KGP2100 from KeyGen Biotech, Nanjing, Jiangsu China). The mucosa proteins within the intestines were isolated with a polyacrylamide substance (Millipore, MA, USA). They were then introduced to polyvinylidene difluoride membranes. These membranes were kept secure and protected for twelve hours, at a temperature of 4°C, after the introduction of the first antibodies. After the introduction of the secondary antibody, they were kept at 25°C, for 120 minutes. The first antibodies (goat polyclonal claudin-1 antibody, *β*-actin rabbit antibody, and rabbit polyclonal ZO-1 antibody) were sourced from Santa Cruz Biotechnology (California, US). The secondary antibodies used were goat anti-rabbit IgG-HRP and rabbit anti-goat IgG-HRP (again, sourced from Santa Cruz Biotechnology).

### 2.6. Statistical Analysis

To evaluate and verify the results, an analysis of variance (or ANOVA) method was used for basic classification and order of a completely randomized design. Before ANOVA, the evenness of its variance was checked using the Bartlett technique. Then, the regularity of the information was tested using the Kolmogorov-Smirnov method. At certain points, a Duncan multiple range method (in accordance with SPSS 22.00 Statistical Software) was utilised to calculate dissimilarities across mean values.

## 3. Results

**Figure 1:**
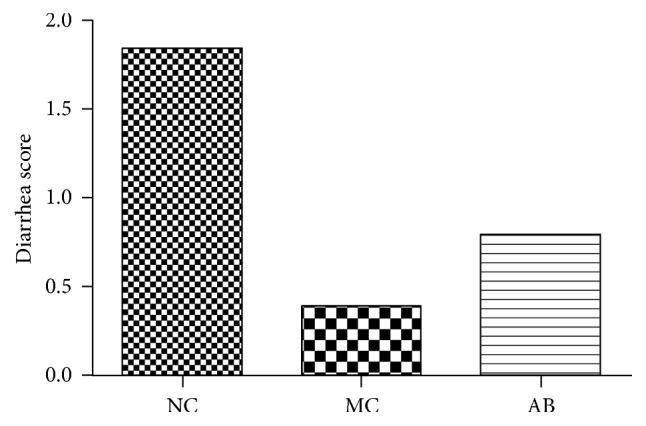
The diarrhea score of pigs provided with the experimental diets described. The experiment lasted for a duration of 28 days.

Figure 1 demonstrates that the diarrhea score for pigs in the MC group was lower than that of the NC group and the AB group. The score for the AB group was still lower than that of the NC group.

Table 2 demonstrates the impact of dietary supplementation with* Macleaya cordata* extract on the serum biochemical parameters and volume of antibodies in the growing pigs on days 7, 14, and 28 after the start of the study.

On day 7, the concentration of IgG was higher (*P* < 0.05) in pigs from the MC group, when compared with the other 2 groups. The serum concentrations of D-lactate, DAO, IgM, and TNF-*α* did not exhibit any substantial or notable differences (*P* > 0.05) across the treatments, on day 7 after the launch of the study.

On day 14, the serum concentration of D-lactate was the only parameter that showed a significant difference (*P* < 0.05) across the treatments. There was a lower concentration in pigs from the AB group. The other serum parameters for the pigs remained the same (*P* > 0.05), due to the impact of dietary supplementation with* Macleaya cordata* extract (Table 2). On day 28, the serum concentration of IgM decreased in the pigs from the MC group, in comparison with those from the AB group (*P* < 0.05). The other parameters exhibited no notable changes (*P* < 0.05), as a consequence of the experimental treatments.

**Figure 2:**
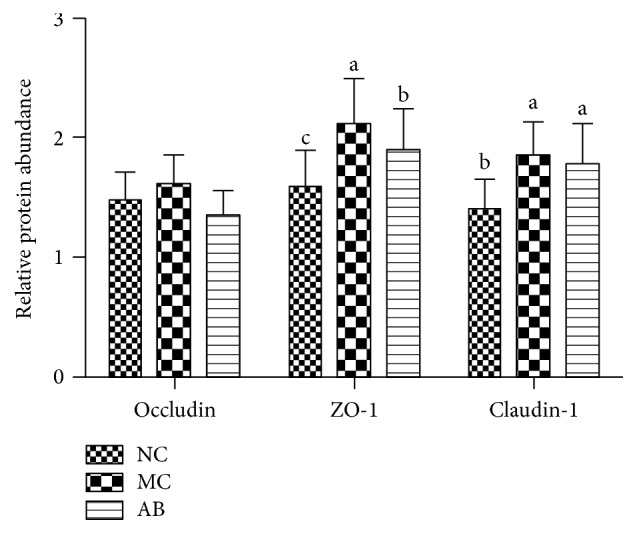
Tight proteins in fairly large volumes, within the jejunal mucosa of pigs. Data is equivalent to ±SEM. The a, b, and c sections, with different letters, are substantially dissimilar to one another (*P* < 0.05). The significance of protein display is the ratio of the densitometry units of tight junction protein and *β*-actin. The study took 28 days to complete; *n* = 6.

Figure 2 demonstrates the protein expression levels of occludin, ZO-1, and claudin-1 within the jejunal mucosa. The occludin levels did not notably differ across any of the groups. The protein expression levels of ZO-1 within the jejunal mucosa increased substantially (*P* < 0.05), in comparison with those in the NC and AB groups. Also, ZO-1 protein increased significantly (*P* < 0.05), in comparison with that of the NC group. Thus, the dietary supplementation of* Macleaya cordata* extract and antibiotics substantially increased the expression of Claudin-1 (*P* < 0.05), particularly when compared with that of the NC group.

## 4. Discussion

Diarrheal syndrome occurs primarily because of an increase in* Escherichia coli* and others pathogenic bacteria inside the gut after weaning. This leads to a loss of water and electrolytes, via the semiliquid faeces [1]. According to Kommera et al. [13] and Kong et al. [1], the supplementation of medicinal plant extracts decreases diarrhea incidence in pigs, mainly because the extract has antimicrobial properties and it aids in the regulation of organic functions, intestinal pH, and peristalsis.

As Newton et al. [7] explain, the antimicrobial properties of sanguinarine have been clearly demonstrated. If it is added to swine diets, as a supplement, it has the potential to support the intestinal colonisation of beneficial bacteria and increase competitive exclusion in GIT. It also reduces water secretion from the intestinal epithelial cells and/or improves the absorption of water and nutrients from the intestinal lumen [1]. These results are primarily caused by an increase in the metabolism of biomolecules and by antioxidant activities within the small-intestinal mucosa.

There is a relationship between intestinal pH, microflora population, and diarrhea incidence in pigs [1]. This is clear because both stabilization of the intestinal pH value at a reasonably low level and the maintenance of an optimal balance of intestinal microbiota play a major role in the reduction of diarrhea incidence [5, 14]. It is noteworthy then that although these key results relating to the the antidiarrheal effect of medicinal plant extracts have been seen in piglets, we also found a similar response in growing pigs.

Crucially,* Macleaya cordata* extract also has other important characteristics, such as its anti-inflammatory effect and immunomodulatory properties [5, 8, 9], which improve intestinal immune function. This reduces inflammation within the small-intestinal mucosa that usually develops as a result of stress. Although the precise mechanisms are not clear, our results demonstrate that* Macleaya cordata* extract, provided as a natural supplement to growing pigs, could be a viable substitute for feed antibiotics.

Serum antibody volume is another useful indicator of humoral immunity. Actually, IgG and IgM are key components and signifiers of humoral immunity in all mammals because they are the major serum immunoglobulins. They protect the extravascular compartment against pathogenic bacteria and microorganisms [15]. Furthermore, IgG also has antibacterial and antitoxin effects [16]. These findings suggest that a higher concentration (*P* < 0.05) of IgG in pigs of MC group (Table 2) leads to an increase in the production of antibodies by B lymphocytes. This is beneficial for immune status and, consequently, animal response [15, 17].

It should be noted that this serum antibody (IgG) did not increase (*P* < 0.05 = on subsequent days (d 14 and 28)), perhaps because of a higher immunological stability in the pigs. However, other studies on medicinal plant additives have shown a variability in the serum concentration of IgG in pigs [15, 17]. Interestingly, the serum concentration of IgM did decrease (*P* < 0.05 = in pigs from MC group). IgM is the first to be synthesised, as a response to infection [17]. Despite variations in the serum concentration of immunoglobulins, after supplementation with* Macleaya cordata* extract, we observed a healthy response in growing pigs (Table 2). According to the work of Tewari et al. [18], the underlying mechanisms that regulate immunological function are likely to be multifactorial. As such, they recommended that future studies use molecular biology and proteomics technologies.

When it comes to sustaining and protecting the translocation of bacteria in the intestines, an undamaged intestinal barrier is very important. It stops allergenic and toxic substances from making their way inside the gut and becoming a danger [19, 20]. D-Lactate and diamine oxidase can usually be found in modest amounts, within the circulation [21]. Yet, if the protective capacity of the intestine is decreased, mucosal permeability begins to rise. This enables a larger volume of D-lactate and DAO to get inside the peripheral circulation. For this reason, D-lactic acid and plasma DAO can potentially be used as indicators of damage to the intestinal barrier system [22, 23]. This paper has discussed, in depth, the relationship between MC extracts and this degree of protection within the intestine. For example, there is much evidence to support the idea that if baby pigs are taken from their mothers and weaned too early, it leads to diminished intestinal barrier capacities [24, 25]. On the other hand, according to the data collected, MC extract supplements actually boost jejunal mucosa DAO. However, it lowers the volume of plasma DAO and D-lactate. This suggests that intestinal mucosal development and barrier strength can be enhanced by introducing MC supplements.

The intestinal barrier is tightly controlled by a carefully arranged piece of the epithelial junctional complex. It is usually called “the tight junction” [26]. The porousness and protective capacities of the intestine have, for a long time, been treated as signifiers of intestinal epithelial barrier strength. It consists of various distinct proteins, like the transmembrane protein occludin [27], some members of the claudin family [28], junctional adhesion molecule [29], various linker proteins such as ZO-1, and more. The most valuable and essential are occludin, ZO-1, and claudin-1 because they are linked to the functional and operational management of the tight junctions [30]. ZO-1 is a valuable linker protein for the tight junction because it joins the C-terminal selections of *β*-actin and occludin [30]. It also functions as a connection between cytoskeleton proteins and the plasma membrane. Claudin-1 seems to be mostly linked to the display of ZO-1 inside the small intestine. Finally, for the epithelial tight junction, occludin is a vital membrane protein. It plays a big part when it comes to sustaining the strength and barrier quality of the tight junction [27]. For this study, we calculated fluctuations within the display of occludin, ZO-1, and claudin-1 at the mRNA level. This allowed us to more accurately judge the molecular catalyst for the decrease in intestinal strength within growing pigs after they had been given artificially increased levels of Zn. The outcomes show that adding* Macleaya cordata* to the diet, as an additional supplement, boosts volumes of ZO-1 and Claudin-1 proteins.

The experiment shows that introducing* Macleaya cordata* extracts to the diet, as an additional supplement, can increase resistance to intestinal damage, to some degree. This is achieved, throughout the growing stage, via increases in the volume and generation of claudin-1 and ZO-1. A number of earlier studies, on animals, had already made it clear that intestinal strength is closely linked to claudin-1 and ZO-1 volumes [31]. According to Kansagra et al. [32], fluctuations within intestinal strength, throughout the weaning stage, can be explained by the deterioration of tight junction reliability and quality. These studies discussed the fact that baby pigs in the weaning phase suffer various pressures (e.g., swapping from a liquid diet to a solid one). This makes them vulnerable to infections created by enterotoxigenic* E. coli* [33]. It happens because tight junction proteins have become disarrayed or their volumes have been lowered [21]. For all of these reasons, epithelial strength is compromised. In turn, this unusually high degree of intestinal porousness leads to increased antigenic vulnerability, a weaker immune system, and persistent inflammation. This greatly decreases barrier strength [34] and, eventually, leads to dangerous levels of after-weaning sickness.

To summarise, boosting the diet with* Macleaya cordata* extract supplements caused a reduction in faecal dysfunction. This result was underscored by larger volumes of claudin-1 and ZO-1. Consequently, the fortifying impact of* Macleaya cordata* supplements may be, to some degree, linked with the enhancement of intestinal barrier strength, particularly when it comes to treating conditions like diarrhea.

## Figures and Tables

**Table 1:** Compositions and nutrient levels in basal diets (as-fed basis).

Ingredients	Content (%)
Yellow corn	74.90
Soybean meal	21.0
L-Lysine HCL	0.23
DL-Methionine	0.07
L-Tryptophan	0.04
L-Threonine	0.10
Poultry fat	1.00
Vitamin premix^1^	1.00
Ground limestone	1.00
Monocalcium phosphate	0.70
Salt	0.30
Total composition	
DM, %	89.70
ME, Mcal/kg	3.38
CP, %	19.40
Ca, %	0.68
Total P, %	0.49
Available P, %	0.21

^1^The trace minerals in the premix provided (per kg diet): 5 mg Cu as CuSO_4_·5H_2_O; 80.5 mg Fe as FeSO_4_·7H_2_O; 0.15 mg I as KI; 0.3 mg Se as NaSeO_3_; 3.3 mg Mn as MnSO_4_·H_2_O; 81.3 mg Zn as ZnSO_4_·7H_2_O; 5 mg vitamin K (menadione); 2.1 mg vitamin B1; 15.2 mg vitamin B2; 30 *μ*g vitamin B12; 5, 110 IU vitamin D3; 400 IU vitamin A; 18 IU vitamin E; and 80 mg choline chloride.

**Table 2:** Effect of dietary supplementation with *Macleaya cordata* extract on D-lactate, IgG, IgM, and TNF-*α* in growing pigs.

Parameter	Dietary supplementation			SEM±	*P* value
	No additive	*Macleaya cordata* extract	Colistin		
*D 7 after initiation of treatment*					
D-Lactate, mg/L	7.95	9.12	7.06	1.119	0.477
DAO, units/mL	2.53	2.46	2.42	0.216	0.536
IgG, mg/mL	138.41^b^	174.45^a^	143.44^b^	10.801	<0.05
IgM, mg/mL	43.20	39.60	38.40	6.635	0.869
TNF-*α*, pg/mL	62.34	58.98	59.36	7.510	0.478
*D 14 after initiation of treatment*					
D-Lactate, mg/L	10.26^a^	8.21^ab^	7.45^b^	0.813	<0.05
DAO	2.42	2.39	2.30	0.166	0.632
IgG, mg/mL	183.89	176.17	184.58	20.04	0.947
IgM, mg/mL	50.00	49.40	51.00	7.799	0.989
TNF- *α*	64.13	59.42	61.37	6.590	0.589
*D 28 after initiation of treatment*					
D-Lactate, mg/L	9.10	9.36	7.60	1.169	0.535
DAO	2.25	2.30	2.18	0.157	0.453
IgG, mg/mL	196.38	219.60	201.72	16.768	0.604
IgM, mg/mL	63.20^ab^	49.60^b^	70.00^a^	6.224	<0.05
TNF-*α*	60.73	58.98	60.22	7.586	0.673

^a,b^Means within the same row with different superscript differ significantly (*P* < 0.05).

The experiment lasted 28 d; *n* = 6.
